# Genetic Admixture and Population Substructure in Guanacaste Costa Rica

**DOI:** 10.1371/journal.pone.0013336

**Published:** 2010-10-13

**Authors:** Zhaoming Wang, Allan Hildesheim, Sophia S. Wang, Rolando Herrero, Paula Gonzalez, Laurie Burdette, Amy Hutchinson, Gilles Thomas, Stephen J. Chanock, Kai Yu

**Affiliations:** 1 Division of Cancer Epidemiology and Genetics, National Cancer Institute, Rockville, Maryland, United States of America; 2 Core Genotyping Facility, SAIC Frederick, Inc., National Cancer Institute - Frederick, Frederick, Maryland, United States of America; 3 Division of Etiology, Department of Population Sciences, Beckman Research Institute and the City of Hope, Duarte, California, United States of America; 4 Proyecto Epidemiologico Guanacaste, San Jose, Costa Rica; 5 Synergie-Lyon-Cancer, Universite Lyon 1, Lyon, France; University of Utah, United States of America

## Abstract

The population of Costa Rica (CR) represents an admixture of major continental populations. An investigation of the CR population structure would provide an important foundation for mapping genetic variants underlying common diseases and traits. We conducted an analysis of 1,301 women from the Guanacaste region of CR using 27,904 single nucleotide polymorphisms (SNPs) genotyped on a custom Illumina InfiniumII iSelect chip. The program STRUCTURE was used to compare the CR Guanacaste sample with four continental reference samples, including HapMap Europeans (CEU), East Asians (JPT+CHB), West African Yoruba (YRI), as well as Native Americans (NA) from the Illumina iControl database. Our results show that the CR Guanacaste sample comprises a three-way admixture estimated to be 43% European, 38% Native American and 15% West African. An estimated 4% residual Asian ancestry may be within the error range. Results from principal components analysis reveal a correlation between genetic and geographic distance. The magnitude of linkage disequilibrium (LD) measured by the number of tagging SNPs required to cover the same region in the genome in the CR Guanacaste sample appeared to be weaker than that observed in CEU, JPT+CHB and NA reference samples but stronger than that of the HapMap YRI sample. Based on the clustering pattern observed in both STRUCTURE and principal components analysis, two subpopulations were identified that differ by approximately 20% in LD block size averaged over all LD blocks identified by Haploview. We also show in a simulated association study conducted within the two subpopulations, that the failure to account for population stratification (PS) could lead to a noticeable inflation in the false positive rate. However, we further demonstrate that existing PS adjustment approaches can reduce the inflation to an acceptable level for gene discovery.

## Introduction

Costa Rica (CR) has a population of about 4 million people mainly comprising two self–described ethnic groups, known as Whites and Mestizos. The recent history of CR suggests that its present population originates from all three major continental populations, namely, Native American, European and African. Such gene flow and admixing scenario provides with a unique resource and a great potential for detecting genes underlying susceptibility to common diseases and traits [1]. The beauty of admixture mapping has been shown in many successful disease mapping projects [2]–[4]. Characterization and analysis of genetic composition of the CR population is critical for future genetic association studies, including genome-wide association studies, based on CR or similar admixed population. A detailed analysis of the underlying population substructure can also yield additional clues regarding the history and geographic distribution of the CR population.

It was reported that the CR population is admixed with 61% European, 30% Native American and 9% African populations on average with variations among different regions in CR [5]. In the Chorotega region, which encompasses Guanacaste, the admixture proportions become 51% European, 35% Native American and 14% African. Most CR studies focused on the Central Valley population and its use in complex diseases mapping investigation [6]. Here we report our detailed population structure analyses based on the data set of 1,301 women from the Guanacaste region of CR genotyped on Illumina InfiniumII iSelect panel containing 27,904 tag SNPs [7].

## Results

### Heterozygosity comparison

**Figure 1:**
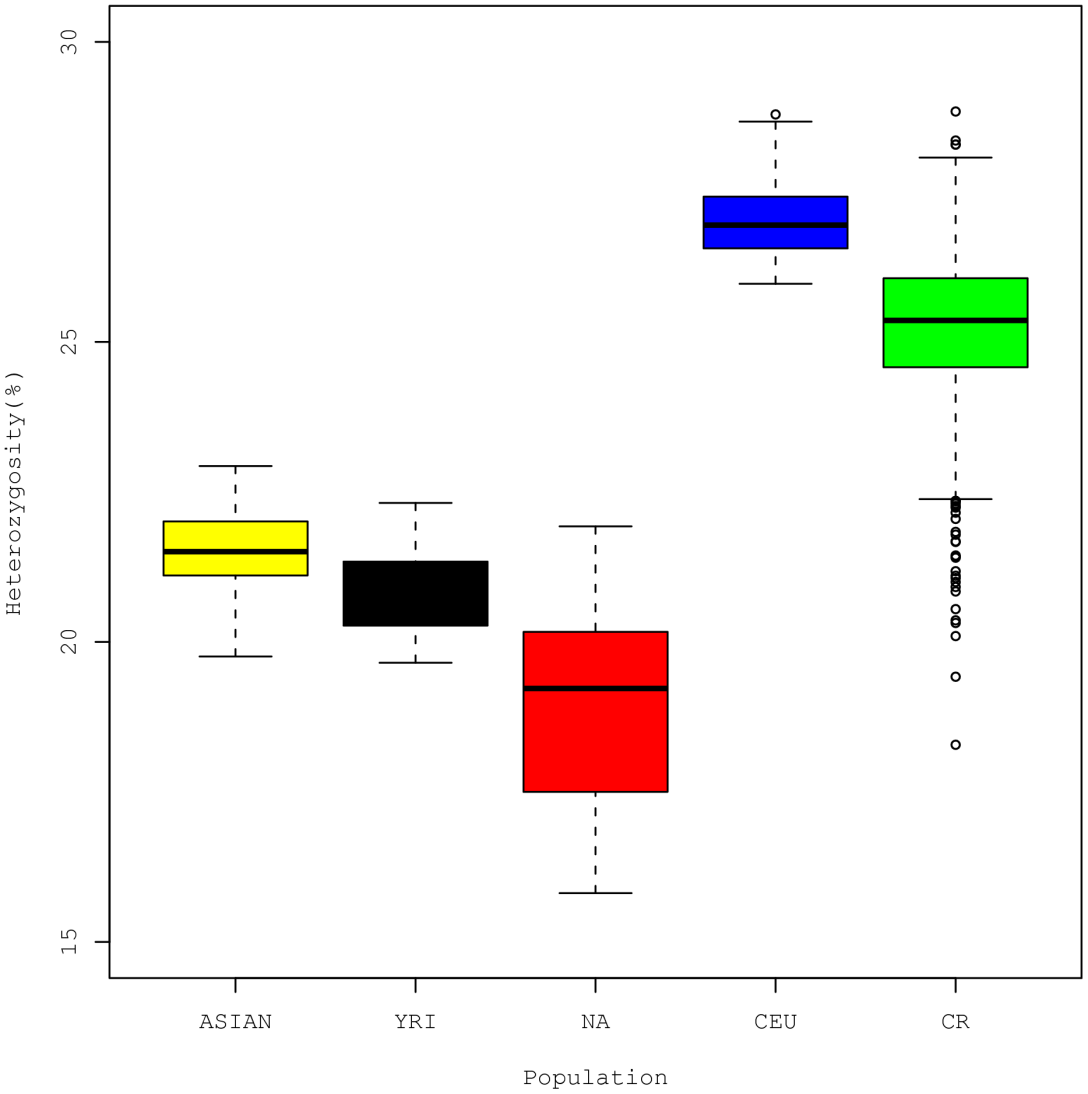
Mean sample heterozygosity by population. Mean sample heterozygosity for each sample was calculated as the number of heterozygote genotypes divided by the total number of non-missing genotypes for the set of 2,663 structure inference SNPs. The sample sizes are 89 ASIAN, 59 YRI, 56 NA, 60 CEU and 1301 CR, respectively.

We evaluated the mean heterozygosity among the four continental reference populations and the CR rural samples over the set of 2,663 structure inference SNPs (Figure 1). It is evident that the pattern of heterozygosity differs among the four reference populations and the CR rural samples. It is notable that the SNPs from the Illumina 550 K were based on a tagging strategy that targeted the SNPs with minor allele frequencies (MAFs) greater than 5% in CEU. Consequently, heterozygosity for the CEU samples is probably overestimated, compared with that expected from a set of randomly selected SNPs. This explains why the mean heterozygosity is the highest for CEU rather than what would have been expected, i.e. for YRI which may not have undergone the population bottlenecks experienced by the “out of Africa” populations [8] and is believed to have the highest genetic diversity. However we did observe that the mean heterozygosity of the CR rural samples is indeed different from that for the CEU, NA, YRI and ASIAN populations. The large variance in the estimated heterozygosity observed in the NA samples could be due to the fact that the NA samples were pooled from three different Native American subpopulations (Figure 1).

### STRUCTURE and principal components analysis

To infer the population structure in the CR population, we applied the STRUCTURE program to the pooled dataset, including the four reference population sets (CEU, YRI, ASIAN and NA) without informing the program which sample was the reference sample. We allowed the program in such an unsupervised mode to infer the underlying ancestral populations as well as the ancestral proportion for each subject, with the number of ancestral populations K fixed at 3, 4, 5, 6, 7, and 8. For a given K, we ran STRUCTURE 10 times with different random seeds (10000 iterations for burn-in phase and 10000 iterations for Markov chain optimization) and recorded *L*(*K*), the log likelihood of the data given *K*, from each run. We used the metric ΔK proposed by Evanno et al. [9] to find the optimal K, which is selected to have the largest ΔK value. As shown in Figure S1, the inferred number of ancestral populations for the pooled data is 4.

**Figure 2:**
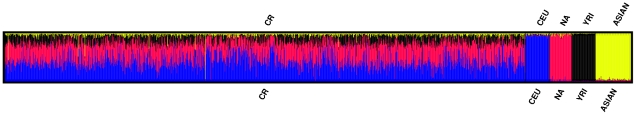
Spectrum plot of admixture coefficients. The admixture coefficient for each sample was the average of 10 independent unsupervised STRUCTURE runs, all with K = 4, which turned out to be the optimal K for the total sample set including CR samples and four continental reference populations (see Figure S1).

Figure 2 plotted average coefficients over 10 independent STRUCTURE runs at K = 4. It indicates that the CR rural samples are derived mainly from a three-way admixture and that the three inferred underlying ancestral populations resemble closely the European, Native American and African reference populations supplied. The overall admixture proportions for our CR rural samples are 42.5%, 38.3% and 15.2% for Europeans, Native Indians and Africans, respectively. The overall Asian component is estimated to be approximately 4%. Except for a few samples with strong Asian origin, we believe that most of the samples have minimal Asian admixture proportion. Overall, our results are consistent with the conclusions of historians that CR was settled mainly by local Native Americans and the influx of both European and West African populations [10].

**Figure 3:**
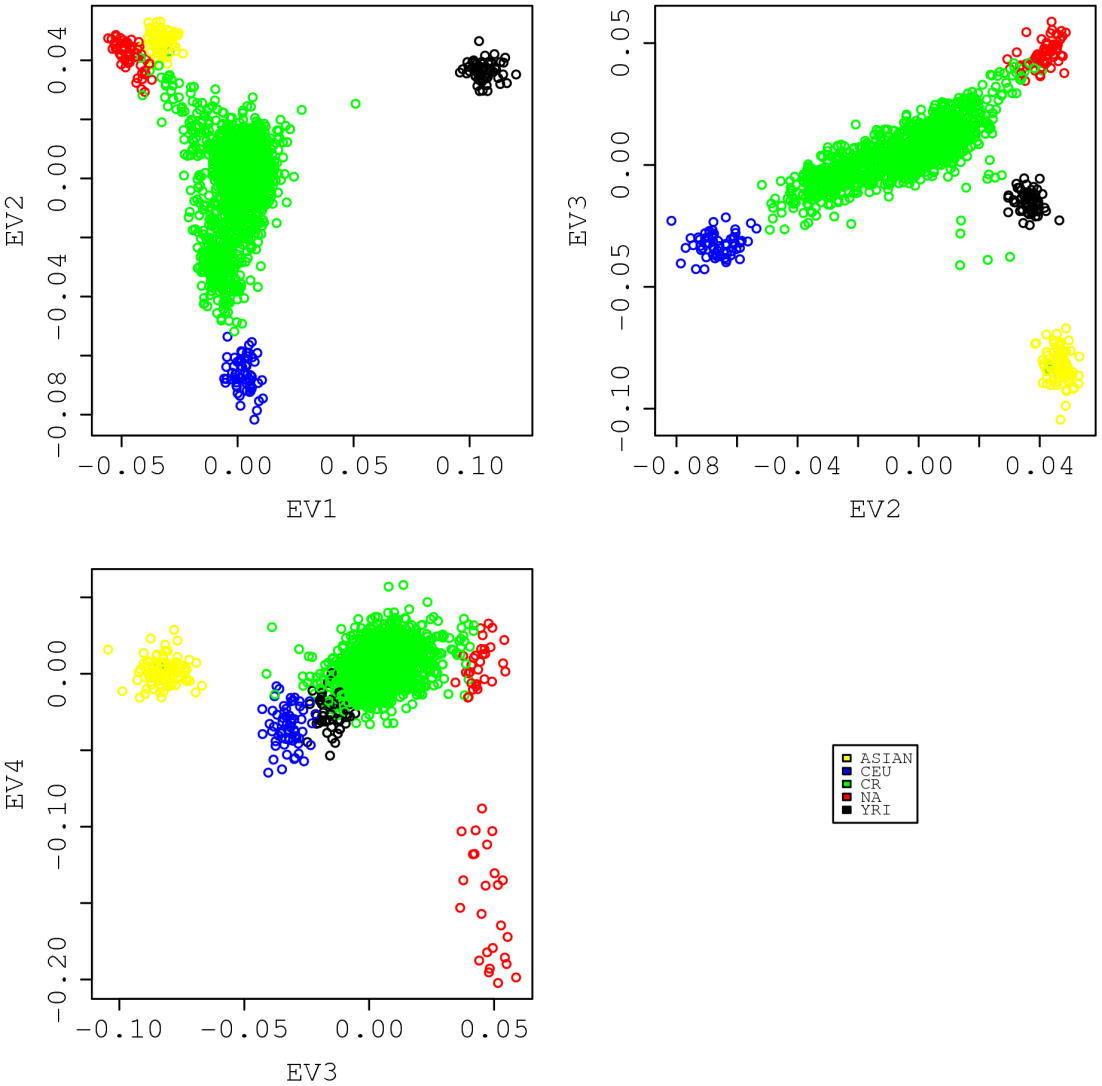
Principal component analysis result. The analysis was conducted on a combined set of samples (the same as the one used for STRUCTURE analysis shown in Figure 2) with 2,663 structure inference SNPs. The top 4 principal components explain a total of 78.5% of the variance in the data, and the corresponding eigenvectors are shown in pairwise scatter plots in this figure.

Principal components analysis was applied to reveal a finer population substructure in the same pooled data used in the STRUCTURE analysis above. Pairwise eigenvector (EV) comparisons are shown in Figure 3. The results again suggest that the CR rural sample is an admixture [11]. In that figure, the dispersed CR rural samples sit in the middle of a plane enclosed by reference populations. It lies mainly along a line joining the two parental populations (CEU and NA), and the line gets pulled and becomes skewed toward YRI (EV2 versus EV1). The Asian population contributes very little to the CR genetic pool other than one obvious Asian sample and six other obvious Asian admixed samples lying between the gravity center of the green CR cluster and the yellow Asian cluster (EV3 versus EV2). The fourth eigenvector separates the Pima Indians from the Colombians and Mayans. The latter would appear to resemble ancestors for CR more closely, since the geographical location of their habitat was closer to Costa Rica whereas the Pima Indians lived mostly in the southern Arizona of the United States (EV4 versus EV3).

**Figure 4:**
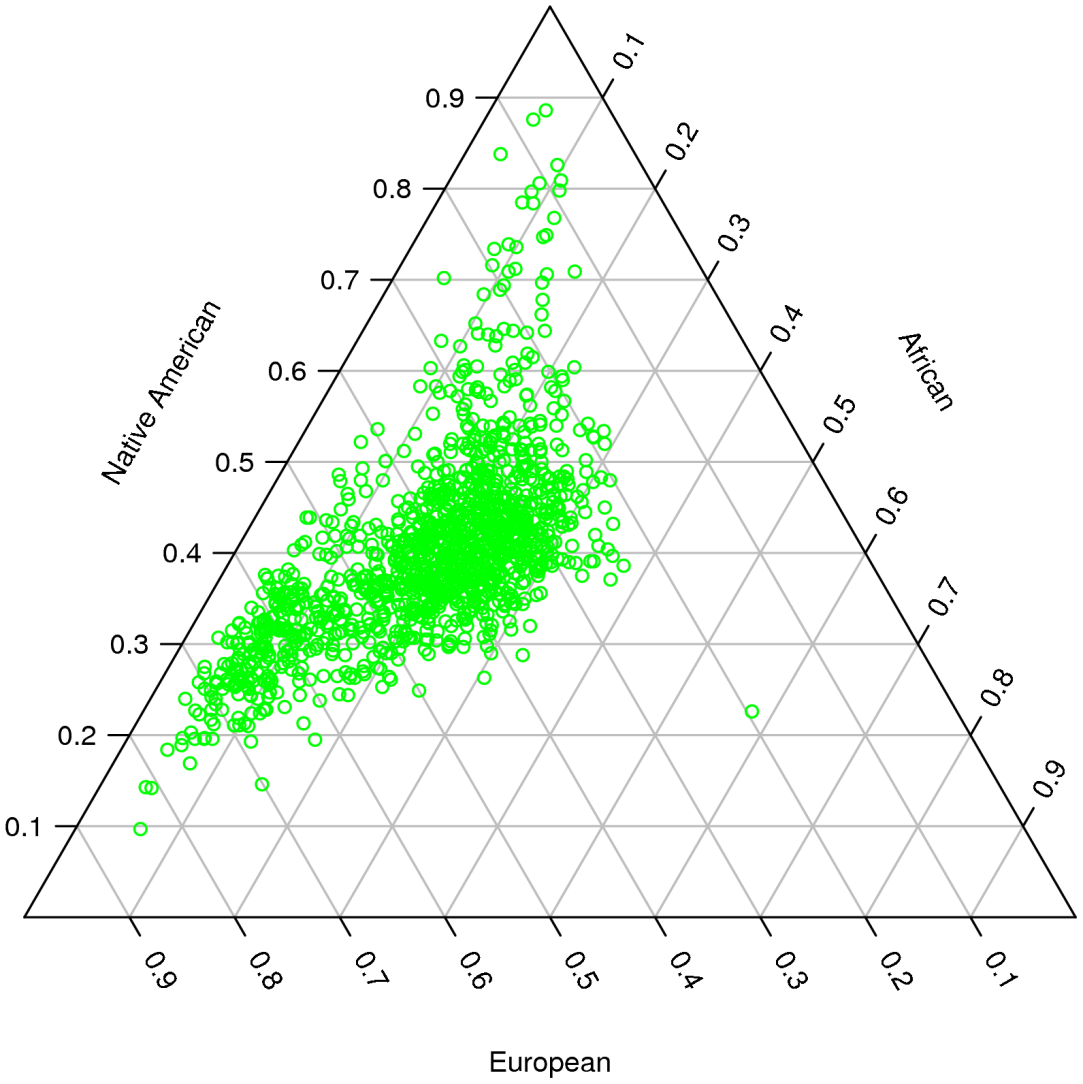
Triangular plot of the admixture coefficients. The supervised STRUCTURE analysis was performed on a refined CR sample set together with three continental reference population sample sets. Coordinates on each axis indicate the admixture proportions from European (CEU), African (YRI) and Native American (Colombian and Mayan) ancestral populations. Each green circle inside the triangle corresponds to a CR sample, and the coordinates on three axes for each sample always add up to 1. The reference samples are at each vortex of the triangle (not shown) because we fixed those as the ancestral populations and inferred three proportion numbers for each CR sample in this analysis.

**Figure 5:**
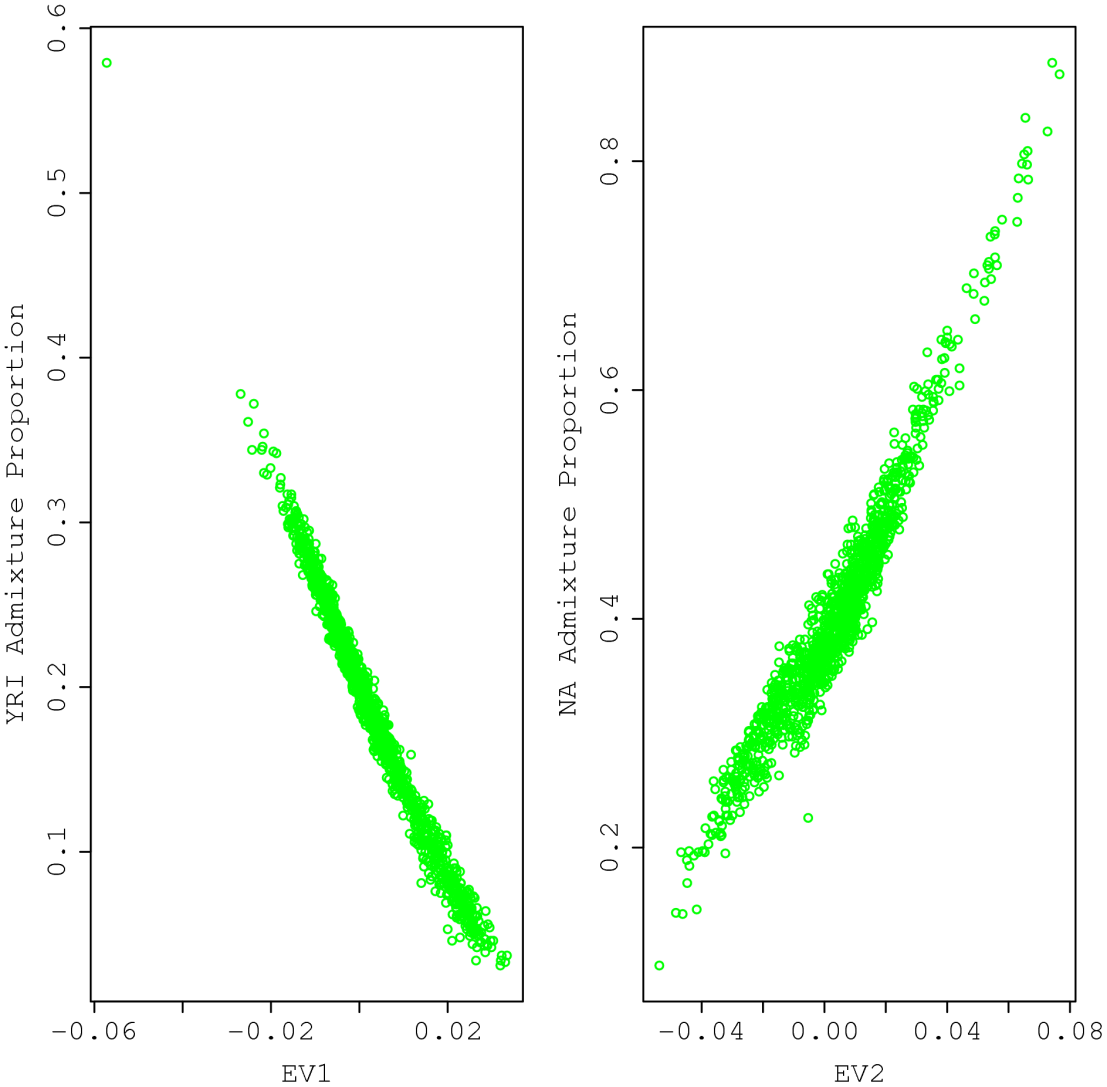
Correlation between STRUCTURE admixture coefficients and PCA eigenvectors for CR samples. Both STRUCTURE analysis and PCA are based on the same data set as detailed in the legend of Figure 4. Pearson correlation coefficients are −0.99 for CEU admixture proportion versus EV1 (left panel) and 0.96 for NA admixture proportion versus EV2 (right panel).

In view of the relatively minor genetic contribution of Asians (<5%) to the CR rural samples, we refined our analysis by removing the 56 samples with Asian admixture proportion greater than 10% and conducted the STRUCTURE analysis on this modified CR rural samples by supplying the program with three reference populations: CEU, YRI and NA. We excluded the Pima Indians but kept the Columbians and Mayans, as suggested by the PCA results shown in Figure 3. The main differences from the initial STRUCTURE run described above are the use of a priori information to fix each reference population and letting the program estimate only three admixture coefficients for the CR rural samples. A triangular plot of the admixture fractions for each subject is shown in Figure 4. The PCA was also performed on the refined CR rural samples together with three reference populations. It is remarkable that the projected values of the CR rural samples on the EV1 and EV2 axes correlated extremely well with their corresponding admixture coefficients estimated by STRUCTURE (Figure 5). Values on the EV1 and EV2 axes correlated well with the YRI and NA admixture coefficients, respectively. Overall, the model-based STRUCTURE and model-free PCA analyses provided consistent results on the degree of admixture of three populations, namely, the CEU, YRI and NA (Colombian and Mayan groups).

The variability measured by the standard deviation of the African admixture coefficient in the CR rural samples was 0.07, which is smaller than the 0.13 observed for the European admixture coefficient. The low variability in the African admixture coefficients observed in the CR rural samples indicates that the major admixing of the African population happened during a relatively early and brief period of time. In this respect, the admixing of African ancestry in the CR rural samples fits the Hybrid-Isolation model [12]. In contrast, it is likely that the larger variablility observed in the European admixture coefficient is due to the admixing pattern of European ancestry in the CR rural samples, which continued for a longer period of time, with the constant influx of new European immigrants. This admixture pattern is consistent with the Constant Gene Flow (CGF) model [12]. The histogram comparing the European and African admixture coefficients is shown in Figure S2. The observed bimodality suggests that there are substructures in the CR rural samples.

### The relationship between population substructure and geographic location

**Figure 6:**
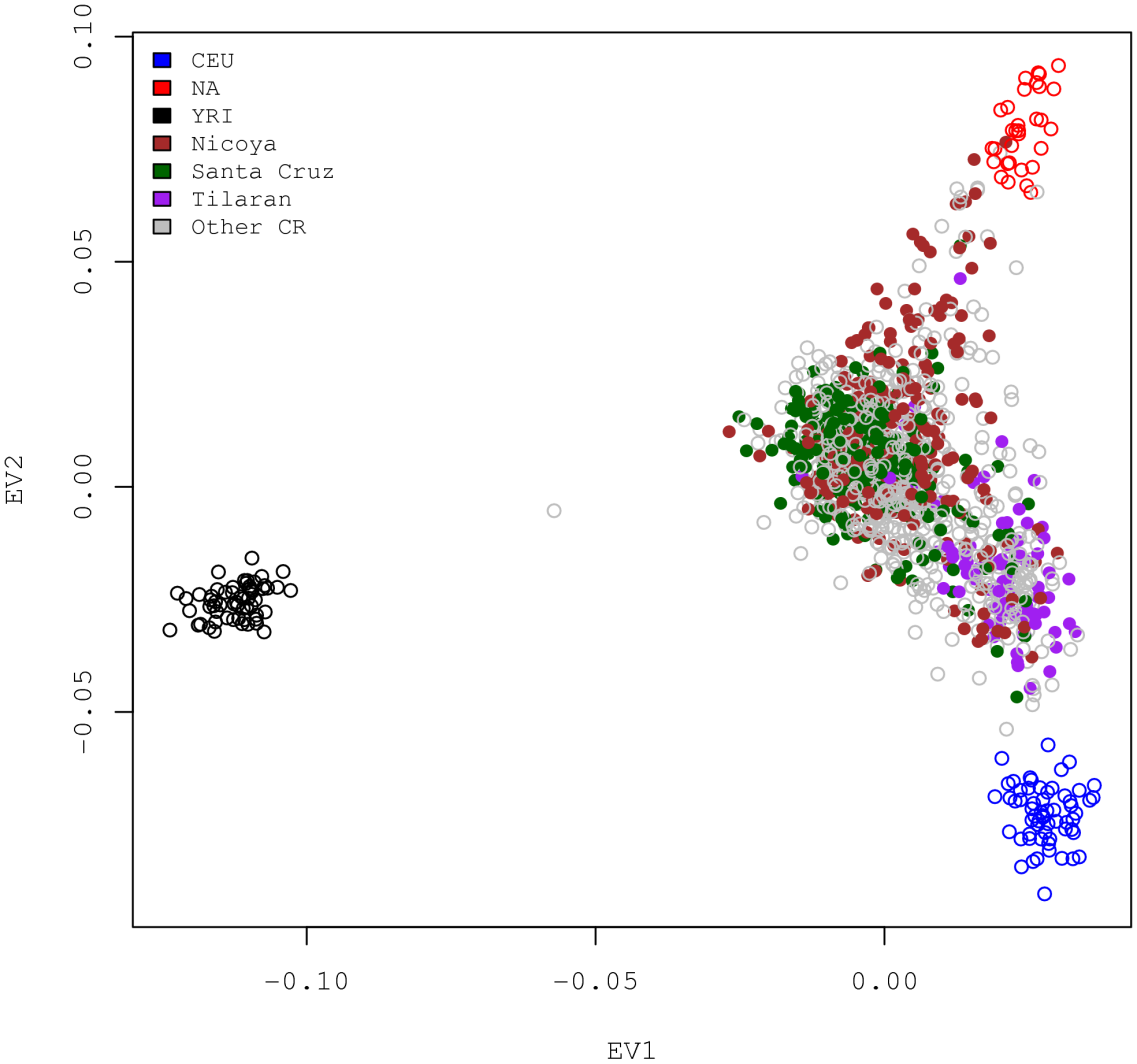
Substructure mirrors geographic location. The principal components analysis was performed on the refined CR samples (excluding 56 with greater than 10% Asian ancestry) together with three reference populations (CEU, NA and YRI). Three cantones (Nicoya, Santa Cruz and Tilaran) in Guanacaste are shown to have distinct distributions in the EV1versus EV2 scatter plot.

It is also interesting to note that the local geographic regions (known as cantones) of the CR rural samples are correlated with their projected positions on the EV1-EV2 plane. We color-coded the subjects in the EV1-EV2 plane according to the cantons from which they were sampled (Figure 6). Two coastal regions (Santa Cruz and Nicoya) and one inland region (Tilaran) are highlighted, using dark green, brown and purple, respectively, in the figure. All other cantons in Guanacaste are included as grey circles in the background, as samples from each of the regions are more or less randomly distributed on the EV1-EV2 plane. Santa Cruz samples (dark green) have larger EV1 values, corresponding to a larger proportion of African ancestry. Some Nicoya samples (brown) co-localized with Santa Cruz, and some samples have larger EV2 values, corresponding to a larger proportion of Native Americans ancestry. Most Tilaran samples (purple) have low EV2 and large EV1 values resembling the CEU samples closely. We found subjects in these three cantons (Santa Cruz, Nicoya, and Tilaran) have significantly different European (or African, or Native American) admixture coefficients (the Kruskal-Wallis rank sum test P-value less than 10^−15^). These results are consistent with demographic data indicating that Nicoya was among the indigenous provinces and was inhabited by several native tribes whereas Tilaran is known to be a more affluent region with a fair number of more recent European immigrants.

### Subpopulation characterization and potential effect of population stratification

Two CR subpopulations could be observed (Figure 4). One group centers around 40% CEU, 40% NA and 20% YRI and the other group centers around 60% CEU, 30% NA and 10% YRI. To evaluate the two subpopulations (characterized as low-CEU or high-CEU admixture) further, we sampled 200 subjects from each subpopulation to form a data set with a total of 400 samples. We used genotypes at the 25,450 SNPs after quality control filtering for the further subpopulation analyses.

To identify SNPs that have different genotype distribution between these two subpopulations, we ran a logistic regression with additive genotype effect between the two subpopulations. The top 10 most differentiated loci are listed in Table S1. Interestingly, the top two loci were in the neighborhood of pigmentation-related genes, such as the rs1426654 SNP within *SLC24A5*, which is an ancestry-informative marker (AIM) with a wide variance in allele frequencies among different populations according to skin pigmentation [13]. Both the *SLC24A5* and *SLC45A2* genes were found in a genome-wide association study to be significantly associated with skin-reflectance measurements [14]. This evolutionarily conserved ancestral allele predominates in African and East Asian populations, whereas the variant allele is nearly fixed due to natural selection in European populations [15]. For the set of 400 samples, we have 137 samples with genotype AA, 81 samples with genotype GG and 182 with genotype AG for rs1426654. The overall frequency for allele A is 57%, which is close to the overall European ancestry for our CR rural samples.

To assess the potential effect of population stratification on association analyses using the CR rural population, we simulated a scenario in which individuals with AA genotypes at rs1426654 were selected as cases and individuals with the other two genotypes were selected as controls. This resulted in 137 cases, out of which 119 (86%) were from the high-CEU subpopulation and 18 from the low-CEU subpopulation, and 263 controls, out of which 81 were from the high-CEU subpopulation and 245 (93%) from the low-CEU subpopulation. We conducted the association test based on a logistic regression model with the additive genotype effect, and observed an inflation factor of 1.76. The inflation factor dropped to 1.014 after the adjustment for the top two eigenvectors (see Figure S3 for Q-Q plot comparison). Although the simulated case represents an extreme scenario, the strong PS effect can still be corrected for adequately by the adjustment for eigenvectors.

**Figure 7:**
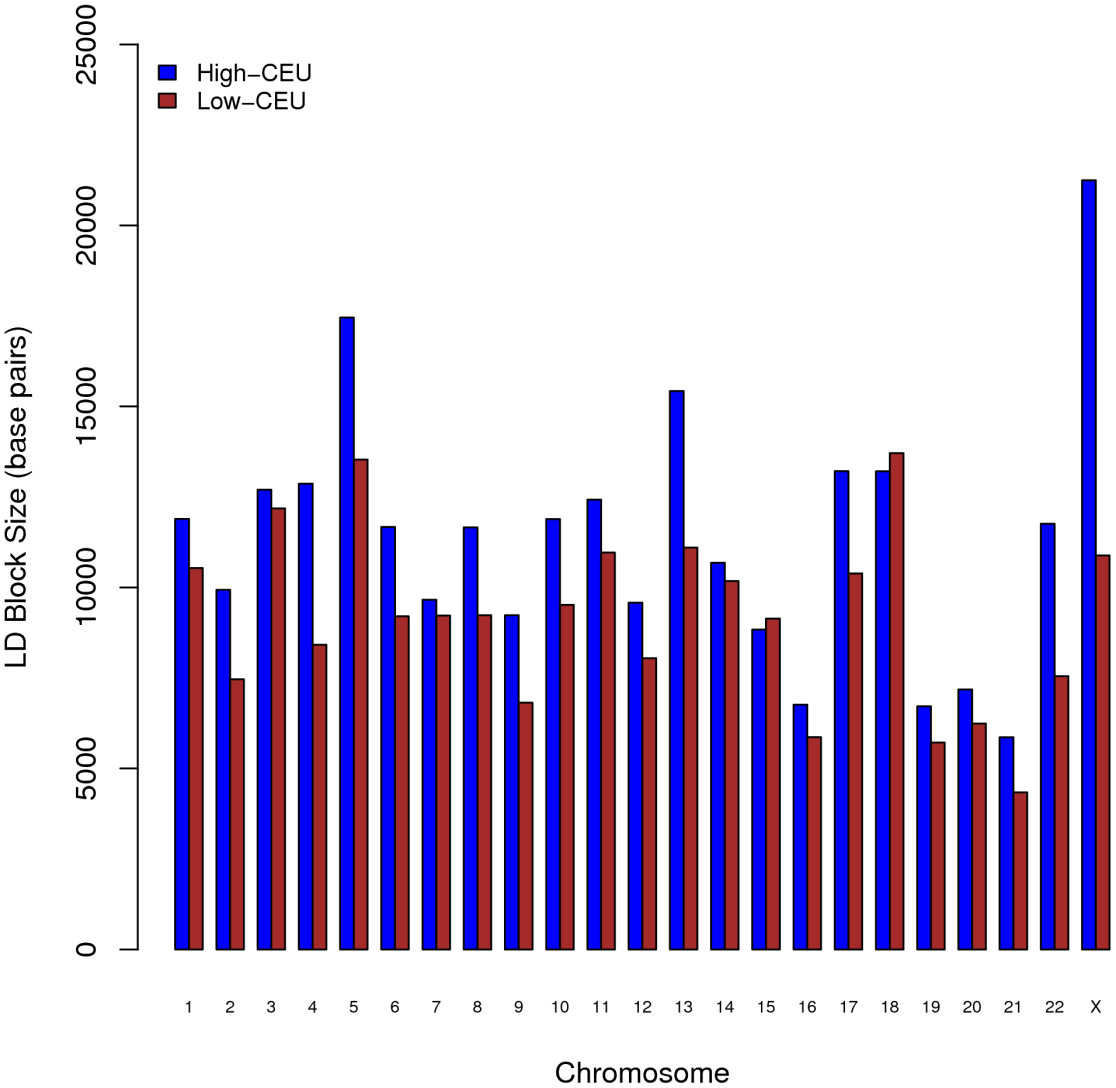
Comparison of the average LD block size for each chromosome between the two subpopulations. LD block size in base pairs for each chromosome was averaged over all LD blocks identified by Haploview^16^. The high-CEU group has average block sizes that were larger than those observed for the low-CEU group for all chromosomes except chromosomes 15 and 18.

Figure 7 compares the average LD block size for each chromosome between the two subpopulations based on Haplotype analysis [16]. The group with the high-CEU admixture had average block sizes that were larger than those observed for the group with the low-CEU admixture for all chromosomes except chromosomes 15 and 18. The overall average block size for the high-CEU group (11 Kb) was 20% higher than that for the low-CEU group (9 Kb). The biggest block for the high-CEU group, 397 Kb, was observed on chromosome 10, whereas the biggest block for the low-CEU group was 273 Kb. This finding is compatible with the hypothesis that the low-CEU group represents an older population compared with the high-CEU group. In this regard, schematically the current CR population was formed by a two-stage admixing process, where the low-CEU group was established first and then served as one of the ancestral populations for the high-CEU group to admix further with CEU. The biggest difference for the average LD block size was observed on the X chromosome, and there was about a 2∶1 ratio between the LD block sizes for the high-CEU and low-CEU groups. It should be noted that the evaluation of block size was limited to a number of selected candidate genes/regions. The block size distribution in other genomic regions may be different.

### LD pattern comparison

**Figure 8:**
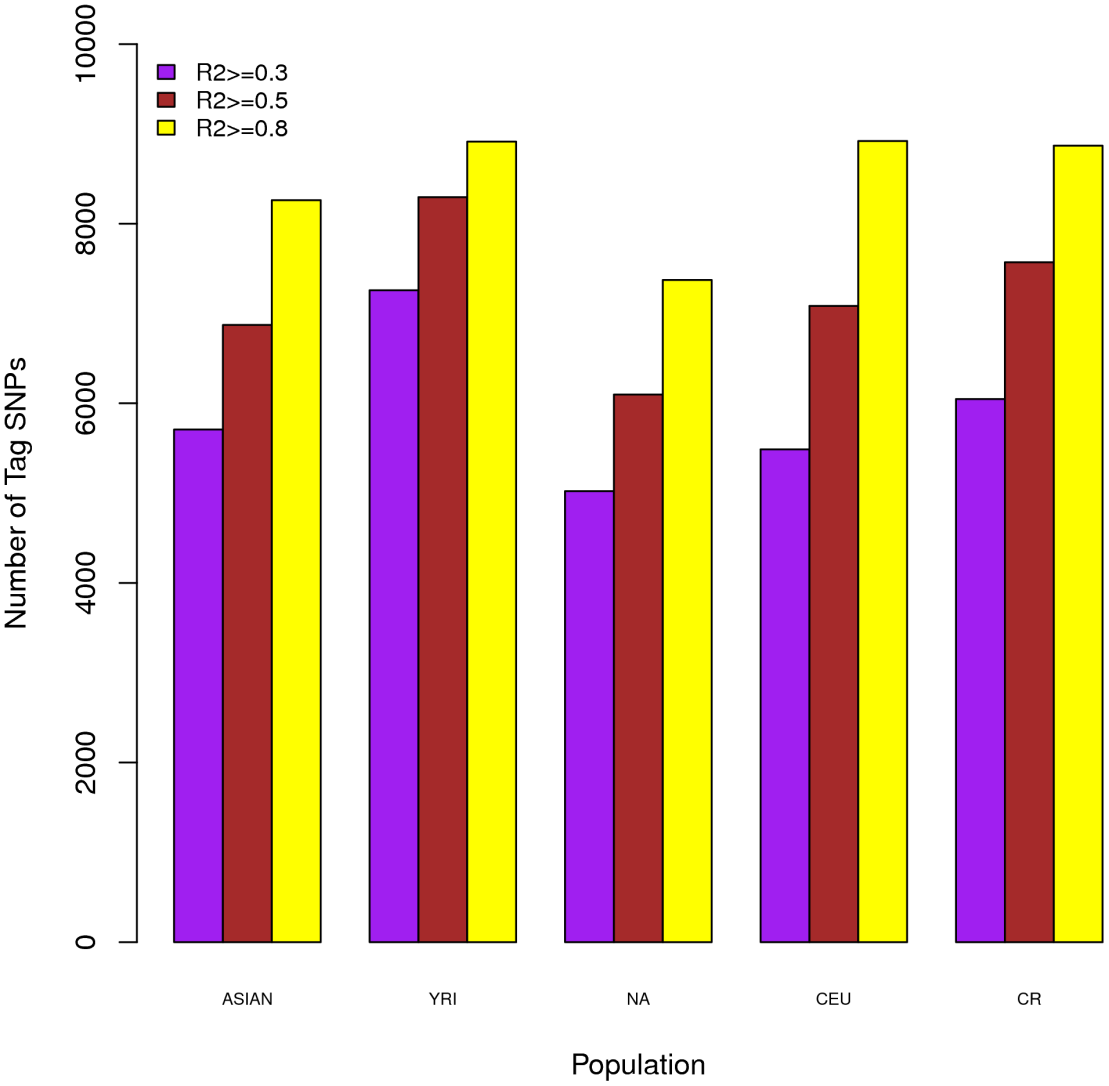
LD comparison among the CR rural population and its ancestral populations. Number of tag SNPs needed to cover the same gene regions was used as a measurement for the strength of LD. Populations with stronger LD would need less number of SNPs tagging the regions given certain r^2^ threshold compared to populations with weaker LD.

Finally we examined the extent of LD observed in the CR samples and compared it with that for continental reference populations based on genotypes measured on the common set of 9,148 SNPs. We ran the LD-tagging procedure implemented in the TagZilla (http://code.google.com/p/glu-genetics/) program, using 500 kb as the maximum between-marker distance, to find the number of tagging SNPs needed for coverage of the whole set of 9,148 SNPs with respect to different pairwise correlations, namely an r^2^ threshold. The results are summarized in Figure 8. Under a given r^2^ threshold, the smaller the number of tag SNPs required, the stronger the LD in that population. Among each of the continental reference populations, it appeared that YRI required the largest number of tag SNPs (i.e., had the smallest LD) and NA required the lowest number of tag SNPs (i.e., had the highest LD) to cover the same genomic region, with CEU and ASIAN falling in between. The magnitude of LD observed in the CR rural samples was stronger than that in YRI but weaker than the ones in the NA, CEU and ASIAN populations. It should be noted that the magnitude of LD observed in the YRI, CEU and ASIAN populations based on the set of 9,148 SNPs may have been underestimated, since this set of SNPs was selected based on a multiethnic tagging strategy targeting these three continental populations.

## Discussion

A thorough understanding of the population substructure of the CR population can provide valuable information for study design, analytical strategies and interpretation of gene mapping projects. Our results show that the Guanacaste region of CR is a heavily admixed population, composed mainly of European (42.5%) and Native American (38.3%) ancestries, with considerable African influence (15.2%). We can not exclude a small influence from Asians (4%). Geographic patterns of the genome admixture in Latin American Mestizos were recently investigated [17] with similar admixture proportions being observed across the board for Latinos.

Recent studies have shown the sex-biased admixture in Latino populations including CR, using mitochondrial DNA (mtDNA) to trace the maternal contribution and Y-chromosome markers to trace the patrilineal input to these highly admixed populations [18], [19]. Genetic variation on X chromosome could also be used as a resource for inferring the sex bias in the admixture [20], [21]. However, we couldn't confirm possible sex-biased admixture in the CR rural sample due to the limited number of independent SNPs on the X chromosome in our genotype data set.

Based on the observed LD comparison with other reference populations, it appears that more SNPs are required to cover the genome for a GWAS study using this CR rural population compared to European or European American population. However this population may be more useful in the follow-up investigation to conduct fine-mapping studies to select the optimal candidate variants for subsequent functional analyses. A more detailed knowledge of the underlying genetic structure in the CR population will require additional genotyping before we can assess the population genetics history even more precisely as well as design and conduct proper gene-mapping studies in CR or a comparable highly admixed population.

## Materials and Methods

### Ethics statement

We have obtained informed written consent from all participants involved in this study. We have obtained ethics approval for this study from the Institutional Review Board at NCI and all involved institutions/hospitals in Costa Rica.

### Study population

We analyzed a total of 1,301 women from Guanacaste, CR, a region located on the Northern Pacific region of CR, with 27,904 SNPs genotyped on a custom Illumina InfiniumII iSelect chip. Those SNPs were selected based on a multiethnic tagging strategy targeting over 1,000 candidate genes/regions in three HapMap reference populations: European (CEU), African (YRI) and Asian (JPT+CHB). The 1,000 candidate genes/regions were selected to investigate a range of cancers, and the data was generated as part of a study by Wang et al. [7], in which they evaluated the association between common genetic variants and risk for human papillomavirus (HPV) persistence and progression to cervical cancer based on a sample of subjects collected from a population-based cohort study of women in Guanacaste, CR [22], [23]. Among those 1,301 subjects, there are 416 cervical intraepithelial neoplasia 3 (CIN3)/cancer cases, 356 HPV persistent cases, 425 random controls, and 104 subjects with histological diagnosis of CIN2. Although these 1,301 subjects were not a random sample from Guanacaste region, the genetic characteristics observed in this large sample should nevertheless give us a relatively comprehensive picture on the genetic landscape of the Guanacaste region population. In the following discussion, we will call this sample set the CR rural samples.

For the continental reference population, we used HapMap (build 23) genotypes including unrelated subjects only (i.e., 60 CEU, 59 YRI and 89 JPT+CHB). We also obtained genotypes from the Illumina iControl database for 56 Native Americans (NAs), including 18 Mayans, 25 Pima Indians and 13 Colombians. All of the 56 NAs were verified by the PREST program [24] to be unrelated individuals.

### SNP selection

After standard quality-control (QC) filtering [7], a stable data set with 25,450 SNPs was generated and made available for further analysis. Subsequently we identified a set of 9,148 SNPs that were common among the iSelect panel for the CR rural samples, Illumina 550 K (used for NA samples) and HapMap SNPs.

To select SNPs with low background LD for the purpose of population substructure inference, we applied a SNP filtering algorithm [25] sequentially on each of the YRI, ASIAN, CEU, NA and CR rural samples and identified a total of 2,663 (29%) population structure inference SNPs, each with an r^2^<0.1 (measured in each of the five sample sets) for any pair of SNPs within a distance of 500 Kb.

### Data analysis

For the population structure analysis, we applied the model based STRUCTURE program [26]–[28] to estimate the admixture proportion for the CR rural samples. The CLUMPP program [29] was used to resolve the label switching and compute the average admixture coefficients. Subsequently the DISTRUCT program [30] was used to plot the spectrum of resulting admixture coefficients for all samples. Complementary to the STRUCTURE analysis, principal components analysis (PCA) [31], [32] was applied to reveal a finer population substructure. Haplotype analyses were performed using Haploview [16] to estimate the average LD block size.

## Supporting Information

Figure S1 Inference of the best K for CR using STRUCTURE. ΔK was calculated as m|L“(K)|/s[L(K)], where m|L”(K)| is the mean of the absolute values of L“(K) averaged over 10 runs and s[L(K)] is the standard deviation of L(K).
(0.12 MB TIF)

Figure S2 Histograms of YRI (left) and CEU (right) admixture proportions for CR samples. The standard deviation for YRI admixture coefficients is 0.07 compared with 0.13 for CEU admixture coefficients. The bi-modality indicates there are substructures in the CR samples.
(0.59 MB TIF)

Figure S3 Q-Q plot of association tests. We observed that adjustment by the top 2 eigenvector could effectively reduce the inflation factor in our simulated test.
(0.39 MB TIF)

Table S1 Loci with different genotype frequencies between the two subpopulations.
(0.03 MB DOC)
