# Supplementary figures and images for: Genetic Admixture and Population Substructure in Guanacaste Costa Rica
Source: PLoS One. 2010 Oct 13;5(10):e13336. doi: 10.1371/journal.pone.0013336 (PMC2954167; doi:10.1371/journal.pone.0013336)

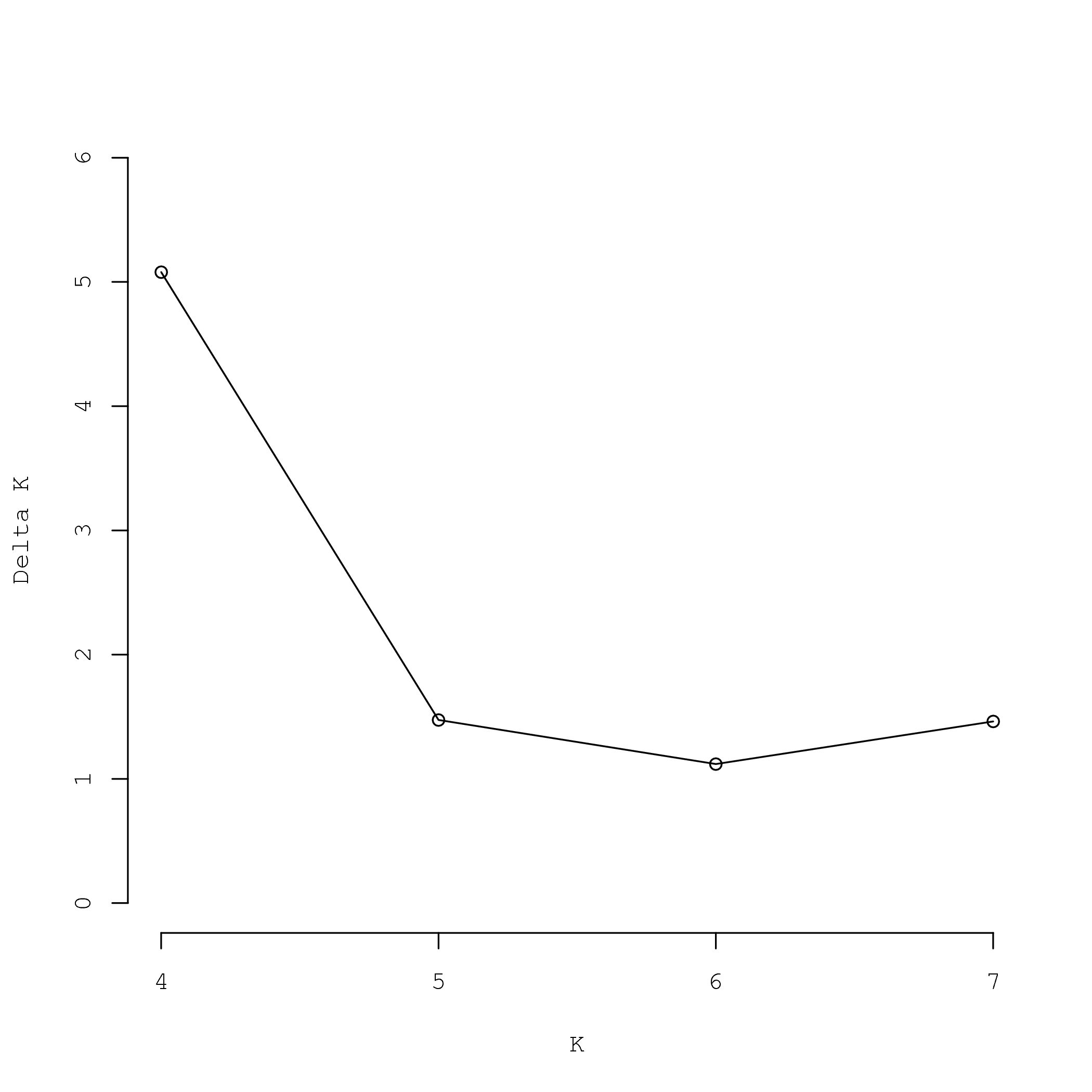

Supplement: Figure S1 — Inference of the best K for CR using STRUCTURE. ΔK was calculated as m|L“(K)|/s[L(K)], where m|L”(K)| is the mean of the absolute values of L“(K) averaged over 10 runs and s[L(K)] is the standard deviation of L(K). (0.12 MB TIF) [file pone.0013336.s001.tif]

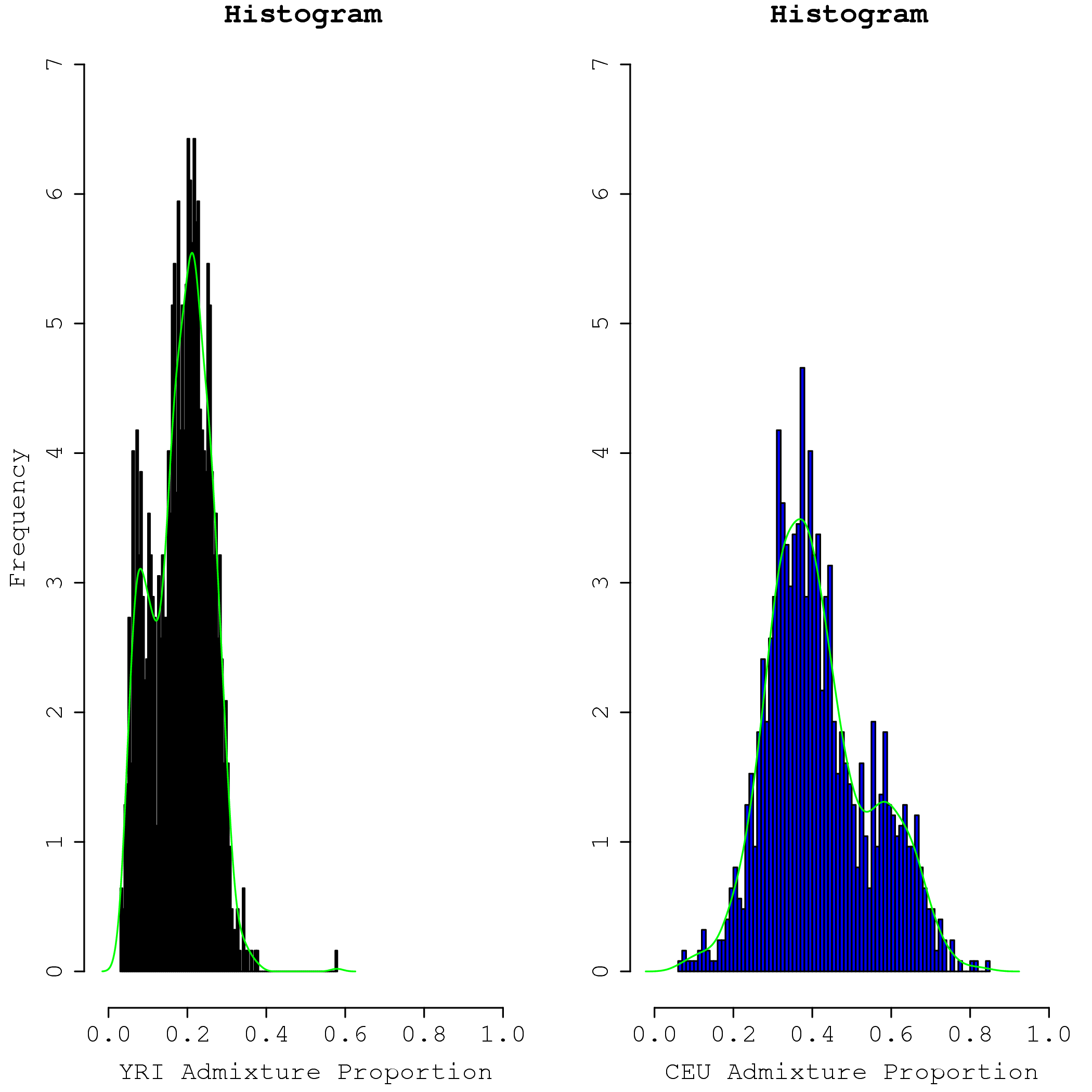

Supplement: Figure S2 — Histograms of YRI (left) and CEU (right) admixture proportions for CR samples. The standard deviation for YRI admixture coefficients is 0.07 compared with 0.13 for CEU admixture coefficients. The bi-modality indicates there are substructures in the CR samples. (0.59 MB TIF) [file pone.0013336.s002.tif]

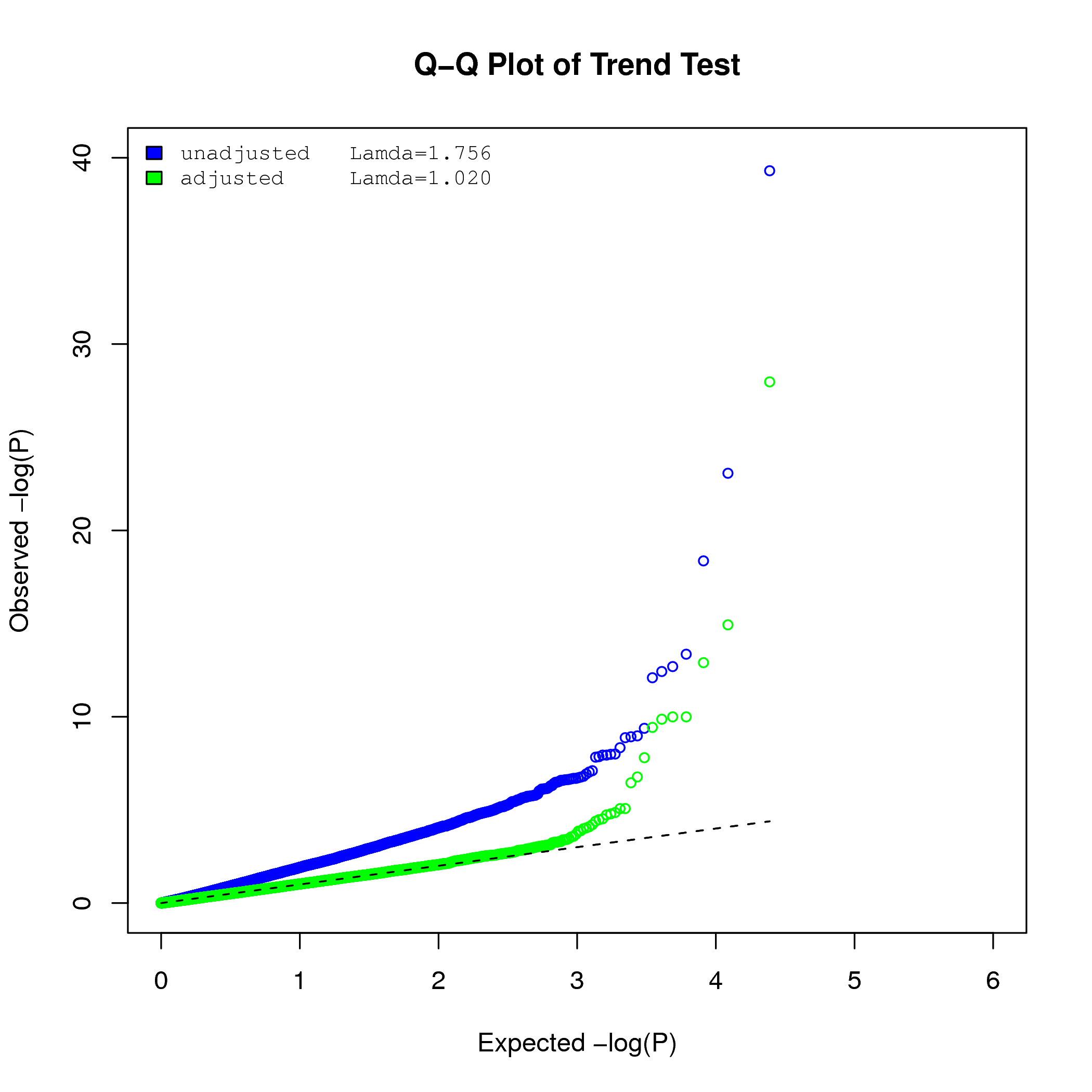

Supplement: Figure S3 — Q-Q plot of association tests. We observed that adjustment by the top 2 eigenvector could effectively reduce the inflation factor in our simulated test. (0.39 MB TIF) [file pone.0013336.s003.tif]
