# Supplementary material for: Genetic Admixture and Population Substructure in Guanacaste Costa Rica
Source: PLoS One. 2010 Oct 13;5(10):e13336. doi: 10.1371/journal.pone.0013336 (PMC2954167; doi:10.1371/journal.pone.0013336)
Supplement: Table S1 — Loci with different genotype frequencies between the two subpopulations. (0.03 MB DOC) [file pone.0013336.s004.doc]

| **Table S1 Loci with different genotype frequencies between the two subpopulations** | | | | | |
| --- | --- | --- | --- | --- | --- |
| **Locus** | **Alleles** | **Trend p-value*** | **CHR** | **LOCATION** | **GENE NEIGHBORHOOD** |
| rs1426654 | A|G | 2.33E-26 | 15 | 46213776 | SLC24A5,MYEF2 |
| rs16891982 | C|G | 4.91E-24 | 5 | 33987450 | SLC45A2,PSMC6P3 |
| rs2424928 | C|T | 4.28E-22 | 20 | 30852297 | DNMT3B,MAPRE1 |
| rs6087990 | C|T | 2.38E-20 | 20 | 30813569 | COMMD7,DNMT3B |
| rs6598443 | T|C | 1.07E-17 | 15 | 99623074 | CHSY1,SELS |
| rs2984296 | C|T | 1.29E-17 | X | 71527286 | HDAC8 |
| rs132792 | C|T | 1.52E-17 | 22 | 40392768 | XRCC6,NHP2L1,LOC100128533 |
| rs6058897 | C|A | 6.34E-16 | 20 | 30861766 | DNMT3B,MAPRE1 |
| rs11637235 | C|T | 9.60E-16 | 15 | 46420445 | LOC100130218,DUT |
| rs7281211 | A|G | 1.91E-15 | 21 | 42618010 | TFF3 |
